# Relationship Between Atopic Dermatitis and Food Allergy in Children

**DOI:** 10.7759/cureus.33160

**Published:** 2022-12-31

**Authors:** Yash Mehta, Darshna G Fulmali

**Affiliations:** 1 Department of Anatomy, Jawaharlal Nehru Medical College, Datta Meghe Institute of Medical Sciences, Wardha, IND

**Keywords:** paediatric age group, allergy test, diet, food sensitivity, eczema, food allergy, atopic dermatitis

## Abstract

Atopic dermatitis (AD) is a chronic inflammatory skin condition characterized by a compromised skin barrier due to a variety of reasons, such as hereditary predisposition, immunological overactivity, and skin microbiome disruption. There is strong evidence linking food allergies (FA) with AD in some children, and many children with AD develop asymptomatic food sensitivity. FA and AD are two frequent childhood illnesses that are closely related. Food allergies affect 30% of kids suffering from moderate and severe eczema and can cause a variety of symptoms, including dry, cracked skin, rash, itchiness, oozing, and crusted skin. While preteens and teens with AD are commonly sensitive to environmental allergens including house dust mites, mold, pollen, or dander of animals, younger kids with AD typically exhibit sensitivity to food items like peanuts, milk, or eggs. A food challenge test (FC) should be used to confirm allergies before recommending a stringent diet that could be hazardous to the patient. While elimination diets continue to be the cornerstone of the management of FA, they should only be carried out under the guidance of a specialist. Topical treatments are crucial for all individuals with AD. Early skin care with emollients, topical steroid treatment, and early introduction of highly allergenic foods are promising methods of alleviating symptoms of AD.

## Introduction and background

Atopic dermatitis (AD), also known as atopic eczema, is a skin disease characterized by long-term inflammation of the skin, resulting in itchy, cracked, swollen, red, and irritated skin. This condition results from the compromised skin barrier of the patient due to a number of reasons including hereditary and genetic predisposition, and immunological pathology. Patients with this condition experience uncontrollable intense itching and irritation of the skin. It is one of the most prevalent chronic inflammatory skin diseases in children. The occurrence of AD is increasing rapid in children with an incidence rate of around 5-20% among the pediatric population [1]. Most of the affected patients usually suffer from mild or moderate cases of this condition; however, severe cases are not uncommon [2]. AD often begins before the age of five years and may continue into the teen and adult years. For many patients, this condition is associated with a relapsing course; the timespan can range widely from a few months to several years. Hay fever, exposure to allergens, and asthma are common risk factors that aggravate the symptoms of AD [3]. In addition, the condition also shows a hereditary trend, with increased chances of a child being affected by AD if either of their parents also suffered from this condition. One or more atopic comorbidities, such as asthma, allergic rhinitis, or food allergies (FA), are inclined to develop in 60% of children suffering from AD [1]. The causes of this phenomenon, known as the "atopic march," are still being investigated.

FA is the body’s exaggerated immunological response to the ingestion of a particular type of food substance that may be fatal to particular individuals. It is characterized by itching, swelling, hives, eczema, and wheezing in mild to moderate cases. In severe cases, there could also be associated difficulty in breathing, anaphylactic shock, light-headedness, and even death. As children grow older, some food allergies are outgrown. However, there is no cure for the condition at present.

Food sensitivity, an unrelated but milder version of FA, involves the presence of food-specific IgE antibodies in blood tests or via skin prick tests without clear clinical reactions. Food intolerance is the nonimmunological reaction towards food, which includes symptoms of diarrhea, gas, bloating, and abdominal pain, but does not have any serious or detrimental repercussions to the health of the patient.

An abundance of evidence and clinical data show that AD and FA and sensitivity are highly interconnected. Children suffering from AD are at a higher risk of food allergies, with IgE-mediated food allergies affecting around one-third of kids suffering from moderate or severe AD [3]. More than 90% of food allergies in children with AD are caused by tree nuts, chicken egg, wheat, peanuts, milk, soy, and fish [4-6]. The most frequent dietary allergies in toddlers and young children are related to hen's egg, soy, cow's milk, and peanut [7]. Older kids commonly show allergies to fish, tree nuts, wheat, and shellfish [4-7]. Birch-related foods have also been reported as probable AD causes in both adults and children [8,9].

At present, there is no cure for AD. Symptoms can only be alleviated by various topical and non-topical treatments, whose main objectives are to strengthen and restore the functioning of the skin barrier, reduce inflammation, and improve quality of life. Usually, by the age of 13 years, after undergoing puberty, most children grow out of this condition. However, the persistence of AD throughout the teenage years and adulthood is not uncommon [6].

## Review

Prevalence and epidemiology

**Figure 1:**
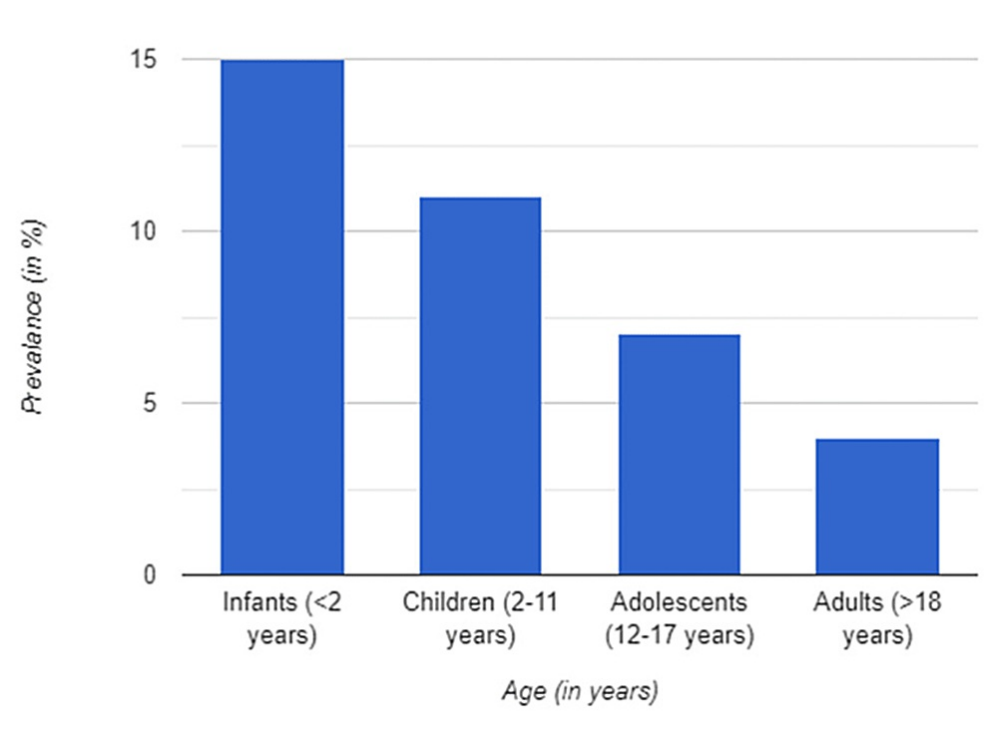
Prevalence of atopic dermatitis by age This figure was created by the author and is not subject to copyright

AD and FA are both prevalent diseases in childhood. Children in the developed world are said to be 3-10% more likely to have AD [10,11]. AD often begins before the age of five years and progresses in a relapsing-remitting manner, with the majority of cases improving spontaneously by adolescence. More than 80% of kids with AD suffer from some mild disease [10,12,13]. The most affected age group is between 0 to 12 years, while the prevalence of the condition in adults is slightly uncommon (Figure 1). While both sexes are affected by AD and FA, the prevalence in male adults is much higher [14,15]. FAs have a deleterious effect on the overall quality of life of the children suffering from this condition, and also their families, despite the very low mortality rate associated with them.

Risk factors and associations

Atopic illnesses run in families for about 70% of AD patients [16]. Children with one atopic parent have an odds ratio for developing AD that is two to three times greater and children with two atopic parents have an odds ratio that is three to five times greater than children with no atopic parents. An elevated risk of getting AD may be linked to a maternal history of AD [17,18].

About 10% of the population of European descent with loss of function due to mutation in the filaggrin (FLG) gene, which encodes an epidermal structural protein, are three times more likely to acquire AD [19]. These subjects had 1 null mutation in the FLG gene. However, around 60% of individuals, despite having the mutation, remain unaffected. Furthermore, many AD patients do not show null mutations for the FLG gene [20-22].

The "western diet" containing large amounts of polyunsaturated fatty acids (PUFAs) and sugar, a small family size, a high level of education, a preference for urban living, and a lack of exposure to UV radiation and low humidity are among the documented environmental risk factors [18]. These elements, along with the individual’s genotype, may change the immunological environment and may enhance the likelihood of developing AD.

Evaluation of atopic dermatitis and food allergy

More than 80% of the kids suffering from AD have mild eczema. There is no need to investigate food allergies if there are no immediate type reactions toward foods. FA may be suspected and ruled out only when AD is still moderate or severe, despite adequate treatment such as emollients and topical creams.

Other manifestations including diarrhea and vomiting can occur in children with AD and FA. Nevertheless, in cases of delayed food-induced eczematic attacks, it may be more challenging to identify the responsible allergen. A thorough clinical history is required, although it might not be able to pinpoint the offending food because AD and other non-IgE mediated reactions can have low sensitivity levels of around 25% [23]. An association with the allergen is more difficult to make because reactions can take hours or even days to manifest. Regular consumption of the offending food increases the risk of chronic symptoms that are harder to pinpoint. The introduction of solid foods or the use of cow's milk formula may be linked to the development of symptoms in young infants. Since a small amount of an allergen may be transferred to the child’s diet through the mother’s milk, there may be a connection between the mother's diet, breastmilk, and AD [24,25]. Asking patients and their parents about their eczema's onset, pattern, and severity, as well as their response to prior therapies and potential triggers including dietary history, allergic reactions, growth, and development, is recommended.

Following an elimination diet can confirm food allergy, with the development of eczematic lesions, and has shown significant efficacy [8]. If reactions are triggered, the FA suspicion is verified, and further controlled, elimination diets without the trigger-inducing item should be followed along with frequent examinations. However, if eczema still persists post-elimination diet, allergies associated with that particular food can be ruled out [26].

Prevention of atopic dermatitis

Daily Emollient Treatment

Since AD usually manifests in the first two years of life, early infancy and the perinatal period have been the principal targets of preventative attempts. In high-risk babies, emollient treatment on a daily basis might reduce the chances of AD. Managing a child struggling with food allergy and AD depends heavily on optimizing skincare, which is inexpensive and important. This condition also has a tendency for remission and relapse which, in the course of time, can get better or get worse without a clear cause. Many parents who have managed AD in their children with the right topical therapy report great improvement or even clearance of eczematic lesions [27,28].

Breastfeeding and Hydrolysed Formulas

Studies linking mothers' food elimination diet during pregnancy and breastfeeding do not show promise for preventing infant AD and FA. Newborn infants are recommended to be breastfed, with an introduction of solid foods into the child’s diet at four months of age. However, regardless of a parent's history of AD, breastfeeding for over 12 months may increase the risk of AD in kids under the age of five [24]. If this is not possible, then hydrolyzed formulas are recommended in high-risk patients [27]. The evidence is still insufficient to draw definitive conclusions or to advise pregnant or nursing women to change their diets or supplement regimens. For children vulnerable to AD, who cannot be exclusively breastfed in the first four to six months of life, certain national and international pediatric societies have recommended the use of hydrolyzed formulas to avoid FA in their guidelines [29]. The EAACI Food Allergy and Anaphylaxis Guidelines Group recently conducted a literature review and came to the conclusion that in infants at high risk of AD, the use of extensively hydrolyzed whey or casein formula for the first four months may have benefits in preventing FA [27]. For further clarity and understanding, definitive and randomized controlled trials are needed.

Pre- and Probiotics and Vitamins

There are numerous ongoing investigations on the use of pre- and probiotics, which have not produced any conclusive findings yet. Probiotics are not suggested as a means of preventing FA by the EAACI Food Allergy and Anaphylaxis Guidelines due to the lack of sufficient data [27]. However, in children suffering from or susceptible to AD, supplementation with vitamin D is recommended, especially if they have low levels. Vitamin D supplementation does not treat or prevent AD but may help certain patients in alleviating certain symptoms like eczematic reactions and skin irritation [30,31]. However, to support this finding, more research spanning longer treatment periods is required.

Treatment

In general, a multifactorial approach is used in the management of AD in children. Emollients are frequently applied as part of topical therapy to help the epidermal barrier heal. Topical corticosteroids and inhibitors of calcineurin, which are approved for use in children older than two years, are other anti-inflammatory treatments that are recommended [32]. Corticosteroids are grouped into seven different classes based on their potency, ranging from class I (very high potency) to class VII (lowest potency), based on which they are prescribed to patients. When the illness is present, these therapies can be used in a reactive manner; however, a dynamic strategy would be to treat the inflammation by applying these medications even if there is no flare [27]. A large number of studies have shown that proactive treatment is directly correlated to a significant decline in sIgE and IgE, thereby reducing the incidence of food allergy [33].

In the short term, topical steroids can be used in combination with bandages and dressings to manage flares in chronic, lichenified AD. Occasionally, despite careful skin care and allergen avoidance, a child's condition will not improve, necessitating systemic therapy or phototherapy. Other general measures include bathing with soap replacements rather than regular soaps and detergents. Some children may be affected by certain triggers, such as extremes in temperature and humidity, wool and synthetic materials, contact allergies, detergents, cutaneous microbial colonization, and food allergens. In these cases, such triggers should be avoided [34]. It is crucial to inform the family of the value of compliance and well-planned education programs.

Prognosis and follow-up

While most kids with an initial allergy to foods like eggs, wheat, soy, and milk eventually learn to tolerate them, some kids' allergies may last until adulthood. Allergies to fish, peanuts, and tree nuts often last throughout adolescence and adulthood. The prognosis for non-IgE-mediated allergies is better overall, with tolerance often developed by the age of five years [10]. When a youngster has outgrown their allergy, the chance should be seized to expand their diet. Mild, well-managed AD also tends to get better over time, but people with severe, poorly-managed disease may require systemic immunosuppressive therapy and perhaps a referral to pediatric dermatology or an allergist [35]. Children suffering from AD or FA are prone to develop asthma and rhinoconjunctivitis; AD and FA are linked to other atopic conditions [36].

## Conclusions

A plethora of research and clinical data indicate the close relationship between AD and food sensitivities and allergies. Food allergies are more common in children with AD and they affect about one-third of children with moderate to severe AD. More than 80% of children with AD also have mild eczema and other such skin irritations. If there are no instant reactions to foods, there is no need to investigate food allergies; only when AD is still moderate to severe despite receiving proper treatment, such as emollients and topical creams, may FA be investigated and ruled out. AD is not curable, at present. Only a variety of topical and non-topical treatments, whose main goals are to improve quality of life, alleviate inflammation, and build and restore the skin barrier, can ease the symptoms. There are a number of options to prevent the prevalence of AD, such as daily emollient treatments, hydrolyzed formulas, pre- and probiotics, and vitamin supplements, to name a few. However, for a permanent viable cure, more extensive research needs to be conducted, spanning various time periods and age groups. The epidemic of AD and resultant FA often leads to many comorbidities such as asthma and rhinoconjunctivitis, since AD and FA are also linked to other atopic conditions.
